# Pediatric systemic lupus erythematosus patients in South Africa have high prevalence and severity of cardiac and vascular manifestations

**DOI:** 10.1186/s12969-019-0382-x

**Published:** 2019-11-26

**Authors:** Michael J. Harrison, Liesl J. Zühlke, Laura B. Lewandowski, Christiaan Scott

**Affiliations:** 1 Faculty of Health Sciences, University of Cape Town, Cape Town, South Africa; 2 Division of Pediatric Cardiology, Department of Pediatrics, Red Cross War Memorial Children’s Hospital, University of Cape Town, Cape Town, South Africa; 3 Division of Cardiology, Department of Medicine, Groote Schuur Hospital, University of Cape Town, Cape Town, South Africa; 4 National Institute of Arthritis, Musculoskeletal, and Skin Diseases, NIH, DHHS, 9000 Rockville Pike, Building 10, 12N248 Room 28, Bethesda, MD 20892-1102 USA; 5 Division of Pediatric Rheumatology, Department of Pediatrics, Red Cross War Memorial Children’s Hospital, Cape Town, South Africa

**Keywords:** Lupus, Pediatric systemic lupus erythematosus, Cardiovascular disease, Echocardiography, Africa, Global health

## Abstract

**Background:**

Pediatric onset of systemic lupus erythematosus (SLE) is associated with major organ involvement, and African patients tend to develop more aggressive disease than patients of European descent. Although cardiovascular involvement is common in pediatric SLE, there are few published reports on the subject. This study describes the frequency and characteristics of cardiac and vascular manifestations of pediatric SLE in a multi-ethnic South African cohort.

**Methods:**

Demographic, clinical, and echocardiographic data were collected from pediatric SLE patients at two centers in Cape Town, South Africa. At the time of investigation, this cohort consisted of 93 participants diagnosed with SLE according to international classification criteria prior to the age of 19. Individuals with cardiac and/or vascular involvement were identified by retrospective chart review. Cardiac manifestations were defined as presence of pericardial effusion, myocarditis, cardiomyopathy, cardiac failure, Libman-Sacks endocarditis, myocardial infarction, and arrhythmia. Vascular manifestations included deep vein thrombosis, pulmonary embolism, sinus thrombosis, stroke, critical limb ischemia, cerebral vasculitis and systemic vasculitis. Statistical analysis was performed using R (v3.4.1).

**Results:**

Cardiac and vascular involvement was present in 47% of the cohort. Previous studies have reported prevalence of 5%—50%. Demographic features of those with cardiac/vascular involvement did not differ from the overall cohort. Echocardiographic data were available for 23 participants. The most common cardiac manifestations were pericardial effusion (*n* = 24) and cardiac failure (*n* = 8), while the most common vascular manifestations were cerebral vasculitis (*n* = 9), stroke (*n* = 7), and pulmonary embolism (n = 7). Cardiovascular manifestations were frequently severe; one third of pericardial effusion cases required intervention, including three cases of cardiac tamponade. Cardiac and vascular involvement conferred an increased risk of mortality (31.1% versus 10.4%).

**Conclusions:**

Cardiac and vascular involvement were highly prevalent in this South African cohort. The mortality rate was high, and severe manifestations were frequent. Prospective research is needed to improve knowledge of pediatric SLE in Africa and to improve outcomes for this high-risk population.

## Background

Systemic lupus erythematosus is a systemic autoimmune disease that can manifest an array of cardiac features including pericarditis, myocarditis, ischemic heart disease, valvular dysfunction, as well as cerebrovascular disease, vasculopathies, and thromboembolic disease [1–3]. Pediatric-onset SLE (pSLE) is associated with greater mortality and a higher rate of major organ involvement in comparison to SLE in adults [4–6]. Although cardiovascular outcomes are not well described in this group, cardiovascular disease is emerging as an important cause of mortality in individuals with pSLE [6, 7]. Cardiovascular manifestations are more frequently reported in patients of African origin, in both adult and pediatric populations [7, 8].

Acute cardiac and vascular presentations of SLE, such as cardiac tamponade and stroke, are associated with significant mortality; however, a greater burden is likely exerted by the chronic cardiovascular effects of SLE in individuals surviving into adulthood [7, 9]. SLE patients develop atherosclerosis earlier and more rapidly than age- and sex-matched controls [10–12]. Early SLE onset confers a high risk of cardiovascular disease in adulthood due to greater cumulative exposure to atherogenic immune dysregulation and multisystem inflammation [9, 13]. There appears to be marked geographical variation in the incidence of cardiac manifestations in pediatric SLE cohorts globally [14–20]. Pericarditis is the most commonly reported cardiac manifestation across the literature. However, its frequency appears to vary significantly across different settings, with individual studies reporting rates of 0.5–36.3%, depending on the setting [7, 16–19]. A large multicenter UK cohort reported only 20% of pSLE presented with serositis (but did not distinguish between pleuritis and pericarditis) and less than 0.01% of patients developed cardiomyopathy, while a population-based study of nearly 300 pSLE patients in the USA reported a diagnosis of pericarditis in 10% of patients [7, 16]. Studies from lower income nations are limited to small case series and case reports. A study of 11 children in Nigeria revealed that 36% had pericarditis, and 45% had left ventricular dysfunction on echocardiography [17]. A study of 33 pSLE patients in Trinidad reported that 24% had pericarditis at presentation and 6% had a diagnosis of stroke [19]. Few published reports on cardiovascular complications of SLE in African populations exist [21–23], although a recent study described high severity of lupus myocarditis in adult SLE patients in Cape Town [24]. A similar study of 50 adult patients in Senegal reported 3 cases of cardiomyopathy and 19 cases of pericarditis, 2 with tamponade physiology [25].

Despite the increased mortality and damage accrual described in pSLE patients, there is a paucity of data on cardiac and vascular manifestations and outcomes in the high-risk population of pSLE patients in South Africa.

## Methods

### Aims, design, and setting

This study was conducted at two large government-funded hospitals in Cape Town, South Africa. Retrospective data were collected by review of medical records. The study was approved by the Human Research Ethics Committee of the University of Cape Town. The objectives of this study were to 1) describe the frequency and characteristics of cardiac and vascular/thromboembolic manifestations of pSLE in a multi-ethnic cohort in South Africa and 2) compare the rates and severity of cardiac and vascular manifestations to a large cohort of pSLE patients in the United States of America (USA).

### Participants

The Pediatric Update on Lupus in South Africa (PULSE) cohort consists of pSLE patients who have received care at either of two tertiary centers (Red Cross War Memorial Children’s Hospital or Groote Schuur Hospital). Most of these patients come from the Western Cape region of South Africa, which has a pediatric population of approximately 19 million children [26, 27]. These patients were retrospectively identified for inclusion in the cohort by searching the digital databases of the two centers using the South African International Classification of Disease (ICD)-10 codes for SLE, lupus, or lupus nephritis from hospital admissions or clinic visits from 1990 to 2018. In addition, on-site physicians were polled to generate a comprehensive list of pSLE patients. The 93 patients registered in the PULSE cohort at the time of investigation were subjected to the following inclusion in our study: age less than 19 years old at SLE diagnosis, who met the Systemic Lupus International Collaborating Clinics (SLICC) [28] or the American College of Rheumatology (ACR) classification criteria [29] for SLE, and had evidence of one or more cardiac or vascular manifestations of SLE. Patients in the PULSE cohort with no evidence of cardiac or vascular disease were included in the analysis for comparison. SLICC and ACR criteria were retrospectively applied to each patient using a predefined checklist during chart review, in order to confirm the diagnosis of SLE for the purposes of the study. Only one patient identified by ICD-10 code was found not to meet SLICC/ACR criteria on chart review and was excluded from the analysis.

### Data collection

Demographic, clinical, and laboratory data were obtained by medical record review. The subject of race in SLE research is relevant, as it has been demonstrated to be an important predictor of disease severity. This study asked patients or parents to identify themselves as belonging to one of five racial groups used in population surveys in South Africa [30]. In South Africa, the term *Black* refers to people of Southern African Bantu-speaking tribal ancestry. The term *Coloured* refers to a racially heterogeneous group of mixed Asian, Malaysian, Indian, Khoisan, Bantu, and European ethnicity. *White* refers to people of European descent; *Indian/Asian* refers to people of Indian or Asian ancestry; and *Other* refers to individuals who do not identify with any one of the described racial categories. A measure of disease activity at initial SLE presentation was obtained using a validated assessment tool, the Systemic Lupus Erythematosus Disease Activity Index 2000 (SLEDAI-2 K) [31]. Clinical manifestations, laboratory results, and medical therapies were captured at the time of diagnosis and over the follow-up period. Autoantibodies including anti-SSA, anti-SSB, anti-RNP, and anti-phospholipid antibodies were not measured consistently enough across the centers to be reported. Medical therapies were recorded as binary variables, with positivity conferred by current or previous use of a medication.

### Cardiac and vascular manifestations

Cardiac involvement was defined as evidence of one or more of the following manifestations: cardiac failure, pericardial effusion, myocarditis, endocarditis (including Libman-Sacks), valvular lesions, dilated or hypertrophic cardiomyopathy, ischemic heart disease, or cardiac arrhythmia. Vascular manifestations were reported separately. These included ischemic or hemorrhagic stroke, venous sinus thrombosis, pulmonary embolism (PE), deep vein thrombosis (DVT), critical limb ischemia, cerebral vasculitis, and systemic vasculitis. Predefined criteria were established for the diagnosis of each manifestation and were classified as clinical, echocardiographic, or radiological criteria. Clinical criteria included physician documentation in the medical record of pericardial effusion; cardiac failure; myocarditis; stroke; critical limb ischemia; or systemic vasculitis. Echocardiographic criteria included evidence of pericardial effusion, structural cardiac remodelling, valvular dysfunction, valvular vegetations, myocarditis, and dilated or hypertrophic cardiomyopathy. For cases of pericardial effusion requiring drainage due to size or causing tamponade, effusion fluid was tested to rule out tuberculosis due to high local prevalence of disease [32]. Radiological criteria included computed tomography (CT) or magnetic resonance (MR) evidence of stroke or venous sinus thrombosis, Doppler ultrasound evidence of DVT, CT angiographic evidence of PE, or MR evidence of cerebral vasculitis. Electrocardiograms could not be reliably located and reviewed for most of the participants, therefore we excluded electrocardiographic data from the analysis.

### Echocardiography

The reports of all echocardiograms performed during the study period for a patient known to have SLE were reviewed. All patients in the PULSE cohort (*n* = 93) were cross-referenced against a repository of echocardiograms at each facility. No echocardiogram reports were available for patients at the Groote Schuur Hospital site prior to 2014. Both transthoracic and transoesophageal echocardiograms were included. The reports for all echocardiograms performed for PULSE patients were reviewed by a pediatric cardiologist. The reports were then summarised as binary variables, with positivity conferred if the feature was found on the echocardiogram report, to allow common key features to be extracted and analysed within the cohort. Cardiac failure was defined as failure of the heart to supply blood to either systemic or pulmonary circulation at an appropriate rate of flow, or to receive venous return at an appropriate filling pressure, resulting in adverse effects on the heart, the circulation, and the patient [33]. Left ventricular systolic dysfunction in children is defined by shortening fraction (SF) < 25% and/or an ejection fraction (EF) < 55% [33, 34, 35]. EF is classified as normal (EF ≥ 55%), slightly reduced (EF 41–55%), moderately reduced (EF 31–40%), and markedly reduced (EF ≤ 30%) [36]. Pericardial effusion is identified in echocardiography as an echolucent space adjacent to the cardiac structures. The major echocardiographic signs that corroborate the clinical diagnosis of cardiac tamponade are systolic collapse of the right atrium and diastolic collapse of the right ventricle, dilatation of the inferior vena cava without respiratory change, and presence of respiratory change in flow velocities through the cardiac valves [37].

### Outcome measures

Outcomes that were measured included mortality and loss to follow up. All patients enrolled in the PULSE cohort who met inclusion criteria were accounted for during the analysis, in one of three categories: actively following up; deceased; or lost to follow-up. The first category encompassed patients actively following up with a healthcare provider, in either the adult or pediatric service. The status of these patients was confirmed using two electronic databases used to manage clinical and attendance information, and laboratory results. Patients were recorded as “actively following up” if they had been admitted, attended an outpatient appointment, or had new laboratory results logged between 1 January 2018 and 31 December 2018. The medical records systems at each center were used to document mortality. Patients were recorded as “lost to follow up” if there was no death documentation, and no record of admission, clinic attendance, or new laboratory results being logged within the previous year.

### Comparison data

Data from a large, recently published, population-based study of cardiovascular disease in pSLE patients in the USA were included for comparison [7]. This study utilised a large administrative database to quantify cardiac involvement in a sample of children and adults with a new diagnosis of SLE between 2000 and 2013. A previously validated algorithm was used to identify a total of 297 children with SLE [38, 39]. The study reported rates of pericarditis, myocarditis, endocarditis, and valvular insufficiency. Secondary outcomes included cardiac tamponade and pericardial drainage. Cerebrovascular disease and venous thromboembolism were reported as covariates of interest. Patients with rheumatic heart disease and infective endocarditis were excluded. This study made use of claims-based data to make inferences about the rate and characteristics of cardiovascular pSLE in a US-based population. The design and context of the study is inherently different to ours, limiting comparative analysis. However, it is the largest report on cardiovascular manifestations of pSLE to date and provides a framework within which our data can be understood in relation to data from another setting. For further comparison, we also included data from several smaller single-center studies which reported cardiac and vascular manifestations in children with SLE [1, 4, 18, 40, 41].

### Statistical analysis

We compared age of SLE diagnosis, race, presenting features, rate of lupus nephritis (LN), laboratory features, SLEDAI-2 K scores, treatment, and outcomes between all patients in the study and the subgroup with cardiac/vascular manifestations. Characterization of the patient population was summarized using descriptive statistics with 95% confidence intervals of means and proportions for cohort comparison. We evaluated enrollment SLEDAI-2 K scores of the cardiac/vascular cohort compared to the entire South African cohort. Where appropriate, Kruskall-Wallis, Pearson, chi-squared, or Fisher’s exact test were used to evaluate differences in both clinical and demographic features and disease scores of the two cohorts. All data was analysed through the statistical package R version 3.4.1 [42].

## Results

A total of 93 pSLE patients were included in the analysis. Demographic features are summarized in Table 1. There were no significant differences in the demographic features between patients with and without cardiac/vascular involvement. The cohort was multi-ethnic, with predominantly admixed ancestry (Coloured; 65.6%) and Black participants (25.8%), reflecting the racial demographics of the region of the Western Cape [36].

**Table 1:** Demographic features of South African pediatric SLE patients

	Cardiac and vascular subgroup (*n* = 45)	Non-cardiac/ vascular subgroup (*n* = 48)	*P*-value
Mean age at diagnosis, years (SD)	11.4 (3.5)	12 (3.2)	0.460
Female % (n)	84 (38)	81 (39)	0.683
Black % (n)	33.4 (15)	18.8 (9)	0.108
Coloured (mixed ethnicity) % (n)	55.6 (25)	75 (36)	0.048
White % (n)	9 (4)	4.2 (2)	0.354
Indian % (n)	3 (1)	2 (1)	0.963

The clinical and laboratory features at presentation are summarised in Table 2. Presenting features were similar between the two groups, except for rates of serositis and positive anti-double-stranded deoxyribonucleic acid antibody (anti-dsDNA). Patients with cardiac/vascular involvement had a trend toward greater disease activity, as measured by the SLEDAI-2 K score. While antiphospholipid antibodies (either anti-cardiolipin antibody or anti-beta-2-glycoprotein antibody) were present at least in one measurement in 10 of the 45 patients, they were not measured in 20 patients with cardiac/vascular disease, or routinely measured in patients without cardiac/vascular disease. The large number of missing data may introduce bias into this evaluation, so we excluded the data from the table.

**Table 2:** Disease activity, clinical and laboratory features of the PULSE cohort

	Cardiac and vascular group (*n* = 45)	Non-cardiac/vascular group (*n* = 48)	*P*-value
Mean SLEDAI-2 K score (SD)	21.2 (7.2)	17.4	0.11
Lupus nephritis % (n)	66.7 (30)	58 (28)	0.407
Arthritis % (n)	58 (26)	64 (31)	0.501
Serositis % (n)	44 (20)	13 (6)	< 0.001
Hypertension % (n)	38 (17)	27 (13)	0.270
ANA positive % (n)	100 (45)	92 (44)	0.117
Anti-dsDNA antibody positive % (n)	93 (42)	79.2 (38)	0.048
Anti-Sm antibody positive % (n)	35.6 (16)	25 (12)	0.267
Median C3 (g/l), range 0.9–1.80	0.90	1.00	0.89
Median C4 (g/l), range 0.1–0.4	0.11	0.10	0.94

*SLEDAI-2 K* Systemic Lupus Erythematosus Disease Activity Index 2000, *ANA* Antinuclear Antibodies, *dsDNA* Double-stranded Deoxyribose Nucleic Acid, *Sm* Smith, *C3* Complement 3, *C4* Complement 4

Thirty-one percent of the PULSE cohort demonstrated cardiac involvement. The rates of each cardiac manifestation observed in the cohort are illustrated in Table 3. In most cases, cardiac involvement was present at the time of SLE diagnosis (84.4%). Notably, three patients presented with cardiac tamponade due to a large pericardial effusion. Approximately one-third of cases of pericardial effusion required pericardiocentesis, and two patients required surgical intervention (pericardial window) due to multiple recurrences.

**Table 3:** Cardiac manifestations in the PULSE cohort

	PULSE cohort cardiac subgroup (n = 29)	Total PULSE cohort (*n* = 93)	Chang et al. (*n* = 297)
Any cardiac manifestation % (n)	100.0 (29)	31.2 (29)	17.8 (53)
Echocardiographicanalysis available % (n)	79.3 (23)	24.5 (23)	NR
Pericardial effusion % (n)	82.8 (24)	25.8 (24)	10.4 (31)
Pericardiocentesis % (n)	27.6 (8)	8.6 (8)	1.5 (4)
Cardiac failure % (n)	27.6 (8)	8.6 (8)	NR
Cardiomyopathy % (n)	17.2 (5)	5.4 (5)	NR
Endocarditis % (n)	0	0	1.0 (3)

*PULSE* Pediatric Update on Lupus in South Africa, *NR* Not Recorded

Vasculitic and thromboembolic manifestations were observed in 29 % of the PULSE cohort; these data are reported in Table 4. Cardiac and thromboembolic/vasculitic manifestations co-existed in 15 % of the PULSE cohort.

**Table 4:** Vascular manifestations in the PULSE cohort

	PULSE cohort vascular subgroup (n = 27)	Total PULSE cohort (*n* = 93)	Chang et al. (*n* = 297)
Stroke % (n)	25.9 (7)	7.5 (7)	4.0 (11)
Venous thromboembolism % (n)	29.6 (8)	8.6 (8)	5.0 (15)
Deep vein thrombosis% (n)	18.5 (5)	5.4 (5)	NR
Pulmonary embolism % (n)	25.9 (7)	7.5 (7)	NR
Cerebral vasculitis % (n)	33.3 (9)	9.6 (9)	NR
Major organ vasculitis other than CNS % (n)	7.4 (2)	2.1 (2)	NR

*PULSE* Pediatric Update on Lupus in South Africa, *NR* Not Recorded, *CNS* Central Nervous System

Echocardiographic data were available for 23 patients, and a total of 92 study reports were reviewed; summarised in Table 5. The most common finding was pericardial effusion, observed in 65% of echocardiographic reports available for review. Of those with effusion, nearly one third required pericardial drainage (pericardiocentesis or surgery). Valvular dysfunction was present in almost half (44%) of those patients who underwent echocardiography. Structural cardiac remodelling and valve status abnormalities were frequently observed. No cases of Libman-Sacks endocarditis were observed.

**Table 5:** Echocardiographic features in the PULSE cohort compared to pSLE patients in the USA

	PULSE subgroup who underwent echocardiography (*n* = 23)	Total cohort (*n* = 93)	Chang et al. (*n* = 297)
Reduced LVEF % (n)	13 (3)	3.2 (3)	NR
Markedly reduced LVEF %	2.2 (1)	1 (1)	NR
Left ventricular hypertrophy % (n)	39 (9)	9.8 (9)	NR
Right ventricular hypertrophy % (n)	13 (3)	3.2 (3)	NR
Right ventricular dilation % (n)	9 (2)	2.1 (2)	NR
Cardiomyopathy % (n)	4 (1)	1.1 (1)	NR
Effusion % (n)	65 (15)	16.1 (15)	10.4 (31)
Tamponade	6.7 (3)	3.2 (3)	0.7 (2)
Effusion requiring intervention % (n)	30 (7)	7.5 (7)	1.5 (4)
Valvular dysfunction % (n)	44 (10)	10.8 (10)	9.1 (27)
Aortic regurgitation % (n)	4 (1)	1.1 (1)	NR
Pulmonary regurgitation % (n)	4 (1)	1.1 (1)	NR
Tricuspid regurgitation % (n)	4 (1)	1.1 (1)	NR
Mitral regurgitation % (n)	4 (1)	1.1 (1)	NR
Multiple valves % (n)	26 (6)	6.4 (6)	NR
Severe valvular dysfunction % (n)	26 (6)	6.4 (6)	NR

*NR* Not Recorded

The outcomes of the patients in this cohort are summarised in Table 6. Mortality rate was significantly higher in the group with cardiac/vascular involvement, while the rate of loss to follow up was similar between the two groups.

**Table 6:** Mortality and loss to follow up in the PULSE cohort

	Cardiac subgroup (*n* = 29)	Vascular subgroup (*n* = 27)	Non cardiac/ vascular subgroup (*n* = 48)	Total PULSE cohort (*n* = 93)
Deceased % (n)	37.9 (11)	29.6 (8)	10.4 (5)	20.4 (19)
Lost to follow up % (n)	13.7 (4)	7.4 (2)	14.5 (7)	11.8 (11)

## Discussion

This study provides the first description of the burden of cardiac and vascular manifestations of childhood-onset SLE patients in sub-Saharan Africa and reports distinct cardiovascular disease severity that may be important to guide the diagnosis and management of these patients. Cardiac and vascular findings were reported in 31.2 and 29.0% of patients in the PULSE cohort, respectively. This is consistent with the reported prevalence (12%—48%) of cardiovascular involvement in pediatric studies [1, 2, 4, 7]. Variation in the prevalence reported in the literature may be explained by single-center design, small sample size, variation in study definitions of cardiovascular involvement, and methodological differences with variable sensitivity for the detection of subclinical manifestations. Greater prevalence of cardiovascular involvement has been described in African-American children with SLE; however, this may reflect overall disease activity rather than an independent risk factor for cardiovascular disease [7, 40]. Most cardiovascular manifestations (84.4%) in our study were evident at initial presentation, which is consistent with other reports [1, 7].

**Fig. 1:**
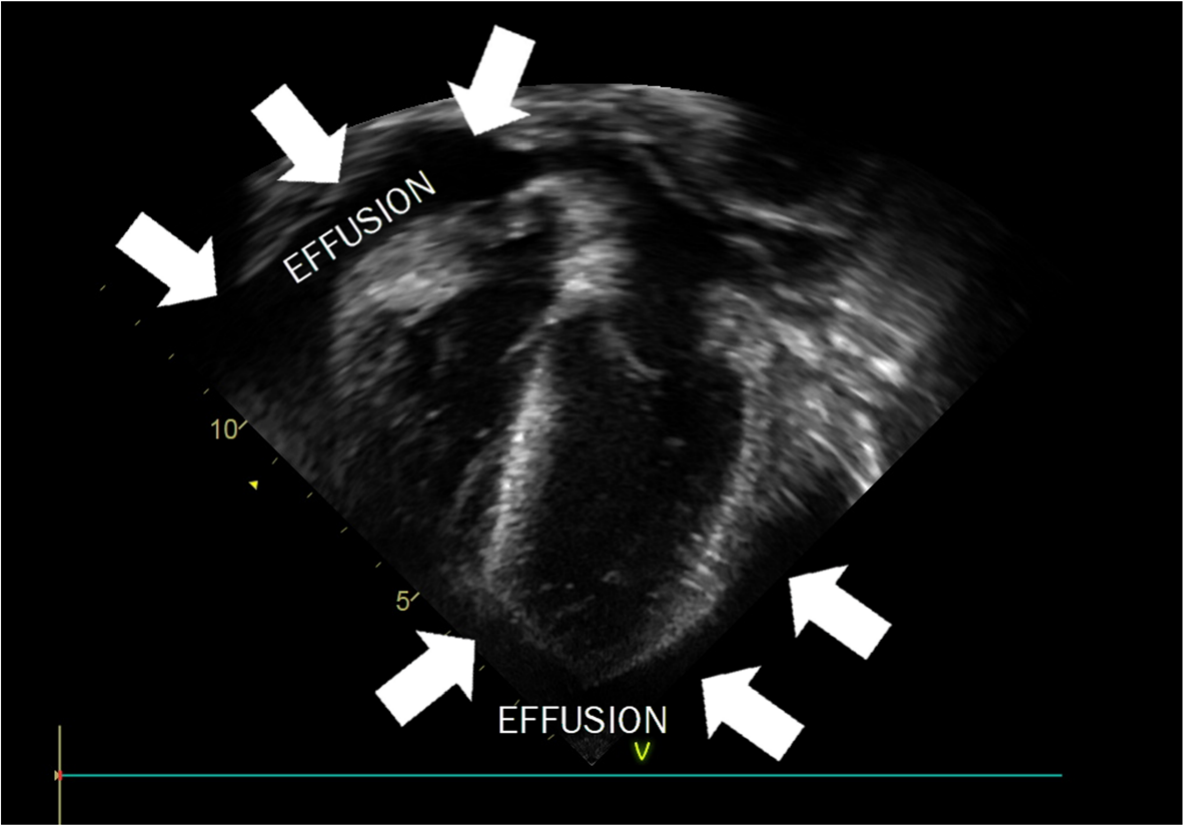
Echocardiogram showing a large circumferential pericardial effusion with diastolic collapse

Pericardial effusion was the most common cardiac manifestation in this cohort, reported at a rate (26.1%) consistent with other pSLE studies [1, 4, 7, 18]. However, the size and impact of these effusions were quite different than other reports. Most notably, there was a high rate of significant effusion requiring intervention in our cohort. Eight patients (8.6%) in the PULSE cohort presented with hemodynamically significant pericardial effusions requiring pericardiocentesis, and there were three cases (3.2%) of cardiac tamponade (Fig. 1.) These manifestations are infrequently reported and relevant literature reporting on tamponade or effusion requiring intervention in pSLE patients is limited to case reports and a single case series [41, 43–46]. However, one analysis of 297 patients with pSLE observed cardiac tamponade in 0.6%, and tamponade required pericardiocentesis in 1.5% [7]. Severe pericarditis requiring intervention is an extreme presentation of pSLE, and was much more frequently reported in the PULSE cohort compared to the USA cohort (8.6% versus 1.5%). The rate is also higher than in other African cohorts. A small Nigerian care series reported none of the pediatric SLE patients with pericarditis having tamponade physiology, and an adult study from Senegal reported 4% of their cohort had tamponade, suggesting a resource-limited setting is not the sole driver of this difference [17]. This finding is consistent with a previous report of a severe SLE phenotype in the PULSE cohort [23].

Echocardiographic data were only available for 23 participants, although 45 presented with cardiac and/or vascular disease. Our findings suggest that routine baseline echocardiogram for all pediatric SLE patients may be of benefit, in order to achieve early detection of cardiovascular disease, although further study is needed for evidence based guidelines for cardiac screening in this setting.

**Fig. 2:**
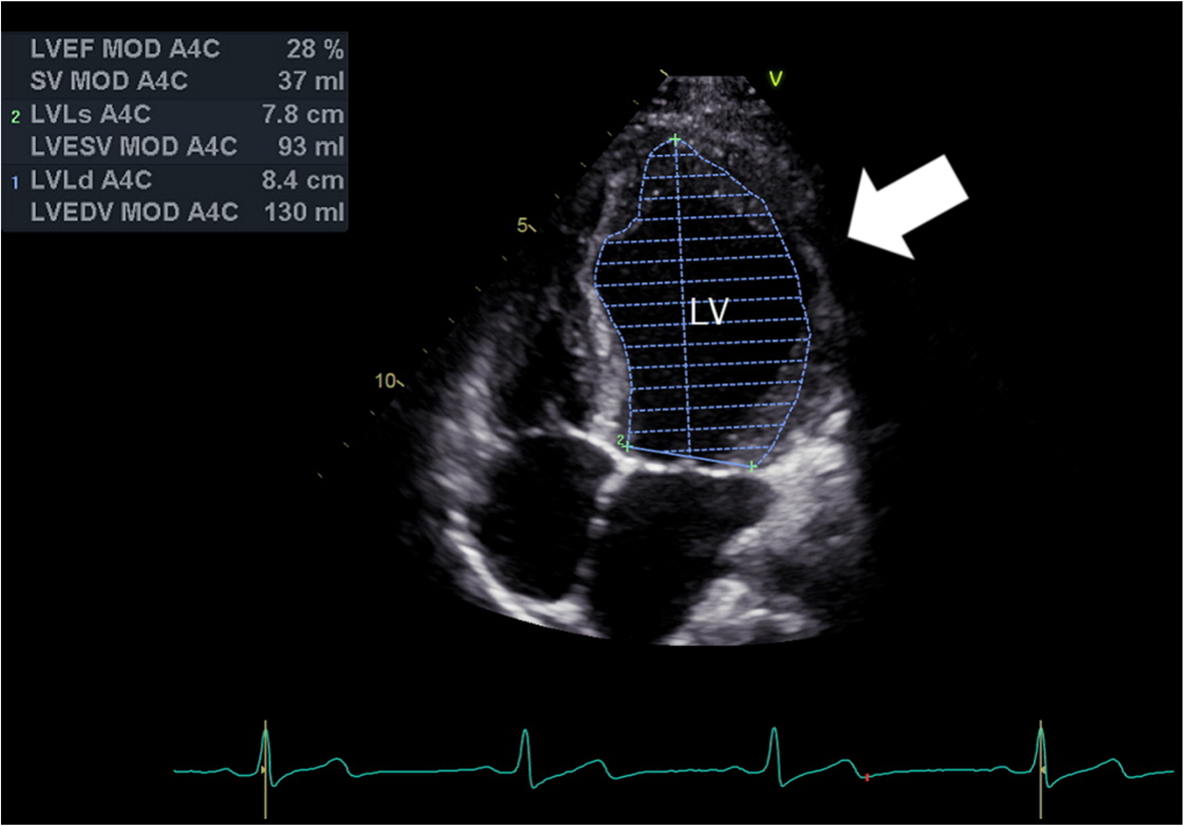
Echocardiogram demonstrating systolic dysfunction with a left ventricular ejection fraction of 28%

In the subgroup of patients who underwent echocardiography, structural cardiac remodelling was highly prevalent, with left and right ventricular hypertrophy being reported in 39 and 13% of cases, respectively. A previously described Taiwanese cohort of 157 patients with pSLE reported structural cardiac remodelling in 17.8% of patients [1]. Valvular dysfunction was reported in 10.8% of the PULSE cohort. This rate is comparable with other studies, which report valve disease at a rate of 6.5 to 19.7% [1, 7, 18, 41]. No valvular vegetations were documented, consistent with the low frequency of this disease reported in the pediatric population [1, 7]. Thirteen percent of patients undergoing echocardiography in this cohort had reduced left ventricular ejection fraction (LVEF) (Fig. 2), suggesting that the rate of cardiac dysfunction in the PULSE cohort is higher than clinically detected. Pediatric SLE patients have been reported to have lower LVEF than healthy age-matched controls that represents cardiac dysfunction even when LVEF is within normal range; therefore, an abnormal LVEF is a concerning finding in our cohort [47]. Studies in both adults and children with SLE demonstrate a high prevalence of subclinical cardiac disease, leading many to motivate for routine use of echocardiography [2, 48–50]. As children with SLE have a higher cumulative risk of damage accrual, and risk factors for cardiac involvement in these patients remain unknown, routine echocardiogram may be of greater benefit in the pediatric population, although formal guidelines regarding the frequency of monitoring are lacking.

Cerebrovascular disease presented in 8.6% of the PULSE cohort, consistent with the rate reported in the literature (2.2 to 12%) [51–53]. This included six cases of cerebral infarction, one case of hemorrhagic stroke, and one case of sinus venous thrombosis. Strikingly, cerebral vasculitis was reported in 9.7% of the PULSE cohort. This rate is high, as cerebral vasculitis is considered a rare entity in both children and adults with SLE [54–56]. Our study was not designed to determine the cause of this high rate in our cohort, but further research is required to investigate and explain this interesting finding.

Finally, the high incidence and severity of both cardiac and vascular disease in this cohort is associated with poor outcomes. Patients with cardiac and/or vascular manifestations had greater mortality compared to those without such involvement (31.1% vs. 10.4%, *p*-value = 0.013).

This study provides initial insights into the burden of cardiac, vascular and thromboembolic disease complications of lupus in a multi-ethnic cohort of children in South Africa. Cardiac and vascular manifestations were common and often severe in the PULSE cohort. In our cohort, echocardiographic data were only available for 51% of patients with cardiac symptoms. Cardiac or vascular involvement was associated with markedly increased mortality compared to peers in the same cohort. This suggests that echocardiography should be performed more consistently in pSLE patients in South Africa. In our opinion, clinicians caring for children with SLE in South Africa should consider performing baseline echocardiography within a year of SLE diagnosis. Additionally, parents and patients should be routinely educated about the recognition and importance of cardiovascular symptoms in SLE. Our study design was not able to identify factors contributing to the high prevalence and severity of cardiovascular manifestations observed in this cohort. In our setting, environmental factors such as poverty, barriers to healthcare, and diagnostic delays may contribute [57, 58]. Equally, our cohort has a high number of patients of African ancestry, and it is possible that genetic factors may play a role in explaining the severe cardiovascular disease observed in this population. The long-term burden of cardiovascular effects of pSLE remains undetermined in the PULSE cohort.

The PULSE cohort is the largest group of pSLE patients on the African continent. However, the relatively small size of the cohort limited statistical power. Participants were recruited from two tertiary medical centers within one city in the Western Cape province of South Africa. Ethnic composition and health systems in this region differ considerably from the rest of the continent, limiting the generalisability of these findings to other settings and populations. The study was further limited by a retrospective design, the documented medical record and the availability of laboratory, echocardiographic and radiological data. Echocardiogram reports at one of the study sites were not reliably stored prior to 2014, limiting the number of participants with echocardiographic data. Electrocardiograms could not be reliably traced for most of the participants, leading the researchers to exclude electrocardiographic data from the analysis. Data for the USA-based comparison cohort was collected between 2000 and 2013, whereas the PULSE cohort collected data between 2005 and 2018. The rate of cardiac disease in children may have differed during these periods.

A large, prospective, multi-site cohort is needed to determine the burden of pSLE in sub-Saharan Africa, to inform practice and improve outcomes for these patients. Translational research is required to investigate genetic markers that confer an increased risk of severe disease in African patients. Prospective studies using echocardiography and non-invasive measures of endothelial function are needed to determine the burden and characteristics of subclinical cardiovascular disease in this at-risk population.

## Conclusions

This study provides initial insights into the burden of cardiac and vascular manifestations of lupus in a multi-ethnic cohort of children in South Africa. Cardiac and vascular manifestations were common and often severe in the PULSE cohort. Prospective studies, long term follow up, and translational research are needed to understand the drivers and long-term implications of these findings.

## Data Availability

The data that support the findings of this study are available from Dr. Chris Scott but restrictions apply to the availability of these data, which contain personal health information, and, therefore, are not publicly available. De-identified subsets of data are, however, available from the authors upon reasonable request and with permission of Drs. Scott and Lewandowski.

## Abbreviations

ACR: American College of Rheumatology
ANA: Anti-nuclear antibodies
Anti-dsDNA: Anti-double-stranded deoxyribonucleic acid antibody
Anti-Sm: Anti-Smith antibody
CT: Computed tomography
DVT: Deep vein thrombosis
EF: Ejection fraction
ICD: International Classification of Disease
LN: Lupus nephritis
LVEF: Left ventricular ejection fraction
MR: Magnetic resonance
PE: Pulmonary embolism
pSLE: Pediatric-onset systemic lupus erythematosus
PULSE: Pediatric Update on Lupus in South Africa
SF: Shortening fraction
SLE: Systemic lupus erythematosus
SLEDAI-2 K: Systemic Lupus Erythematosus Disease Activity Index 2000
SLICC: Systemic Lupus International Collaborating Clinics
USA: United States of America
